# Differences in the Kinetic of the First Meiotic Division and in Active Mitochondrial Distribution between Prepubertal and Adult Oocytes Mirror Differences in their Developmental Competence in a Sheep Model

**DOI:** 10.1371/journal.pone.0124911

**Published:** 2015-04-20

**Authors:** Giovanni Giuseppe Leoni, Maria Grazia Palmerini, Valentina Satta, Sara Succu, Valeria Pasciu, Angelo Zinellu, Ciriaco Carru, Guido Macchiarelli, Stefania Annarita Nottola, Salvatore Naitana, Fiammetta Berlinguer

**Affiliations:** 1 Department of Veterinary Medicine, Sassari University, Sassari, Italy; 2 Sardinian Animal Biodiversity Center (Centro di Competenza per la Biodiversità Animale-CCBA), Sassari, Italy; 3 Department of Life, Health and Environmental Sciences, L’Aquila University, L’Aquila, Italy; 4 Department of Biomedical Sciences, Sassari University, Sassari, Italy; 5 Department of Anatomy, Histology, Forensic Medicine and Orthopaedics, La Sapienza University, Rome, Italy; Institut de Génétique et Développement de Rennes, FRANCE

## Abstract

Our aim is to verify if oocyte developmental potential is related to the timing of meiotic progression and to mitochondrial distribution and activity using prepubertal and adult oocytes as models of low and high developmental capacity respectively. Prepubertal and adult oocytes were incorporated in an in vitro maturation system to determine meiotic and developmental competence and to assess at different time points kinetic of meiotic maturation, 2D protein electrophoresis patterns, ATP content and mitochondria distribution. Maturation and fertilization rates did not differ between prepubertal and adult oocytes (95.1% vs 96.7% and 66.73% vs 70.62% respectively for prepubertal and adult oocytes). Compared to adults, prepubertal oocytes showed higher parthenogenesis (17.38% vs 2.08% respectively in prepubertals and adults; P<0.01) and polispermy (14.30% vs 2.21% respectively in prepubertals and adults; P<0.01), lower cleavage rates (60.00% vs 67.08% respectively in prepubertals and adults; P<0.05) and blastocyst output (11.94% vs 34.% respectively in prepubertals and adults; P<0.01). Prepubertal oocytes reached MI stage 1 hr later than adults and this delay grows as the first meiotic division proceeds. Simultaneously, the protein pattern was altered since in prepubertal oocytes it fluctuates, dropping and rising to levels similar to adults only at 24 hrs. In prepubertal oocytes ATP rise is delayed and did not reach levels comparable to adult ones. CLSM observations revealed that at MII, in the majority of prepubertal oocytes, the active mitochondria are homogenously distributed, while in adults they are aggregated in big clusters. Our work demonstrates that mitochondria and their functional aggregation during maturation play an active role to provide energy in terms of ATP. The oocyte ATP content determines the timing of the meiotic cycle and the acquisition of developmental competence. Taken together our data suggest that oocytes with low developmental competence have a slowed down energetic metabolism which delays later development.

## Introduction

Oocyte quality is currently regarded as the key limiting factor in female fertility, and it is referred to as its intrinsic developmental potential. It depends upon the biochemical and molecular state that allows a mature oocyte to be fertilized and develop to an embryo, which upon transfer will enable healthy development to term. In accordance with this, poor oocyte quality results in either aneuploidy, epigenetic disorders, arrested embryonic development or spontaneous abortion [1–3] One of the great challenges that remain in the fields of reproductive biology and medicine is to understand the nature of the molecular and cellular processes that control oocyte quality. The process by which mammalian oocytes acquire their developmental competence involves complex and distinct events of both nuclear and cytoplasmic maturation. Nuclear maturation mainly involves germinal vesicle break down (GVBD), chromosomal condensation (metaphase I—MI) and segregation, and polar body extrusion (metaphase II- MII). Cytoplasmic maturation involves organelle reorganization, increase in the content of calcium stores, and storage of molecules that act in the overall maturation process, fertilization and early embryogenesis [4].

The best test to determine oocyte quality is the in vivo transfer of the obtained embryo, followed by gestation, birth, and continuing health of the resulting offspring. Obviously, ethical problems in human reproductive technologies restrict these experimental approaches to animal models. However, these experiments are cost prohibitive, take a great deal of time to achieve results, and require large animal numbers to obtain data that can be statistically analyzed. Thus, other parameters can be used to assess oocyte quality. Endpoints that are usually used for this purpose include nuclear maturation success (to MII), fertilization rates, kinetic of embryonic development, development to the blastocyst stage, blastocyst total cell number, inner cell mass and trophectoderm cell numbers, oocyte and embryo metabolism, ATP content, mRNA and proteins storage, and mitochondrial activity and distribution. Examination of all of these parameters will assist in discovering the complex mechanisms that synchronously act to impart oocyte quality.

Cytoplasmic maturation is associated with a considerable increase in storage of foodstuffs and informational macromolecules such as mRNAs, proteins and transcription factors. At this time, the oocyte undergoes a regional redistribution of cytoplasmic organelles which is functional to the acquisition of meiotic competence that ultimately will influence the basic properties of the future embryo [4, 5].

Increasing evidence shows the role of mitochondria as determinants of developmental competence for mammalian oocytes, due to their function as energy suppliers [6]. The quantity and functional status of mitochondria contribute to the quality of the oocyte and play important roles in fertilization and embryo development [7]. In particular, it has been shown that ATP synthesized by mitochondria is crucial for protein synthesis and phosphorylation, which in turn represents a fundamental requirement for progression of oocyte maturation [8]. Reduced efficiency of mitochondrial respiration and ATP content in the oocyte has been shown to be related to poor embryo development [8–10].

In addition, it has been postulated that kinetics of cell cycle could be used as an indicator of oocyte and embryo quality. Cleavage kinetics has been related to the quality of blastocysts in several species (human [11]; porcine [12]; bovine [13]; ovine [14]). Dominko and First [15] firstly positively related the kinetic of MII to the cleavage and blastocyst rates, and timing of meiotic progression is considered as a marker of oocyte quality [16]. However, information on factors affecting the kinetic of meiotic cycle in mammalian oocytes is lacking.

Comparative analysis of oocytes with high and low developmental potential is essential to establish reference data that could indicate important mechanisms involved in the acquisition of developmental competence. The follicular origin of the oocyte has a significant impact on its developmental potential and it appears that once the oocyte is removed from its follicle its intrinsic developmental potential is determined [17]. Although several studies have reported successful in vitro embryo production and the birth of live offspring from oocytes of prepubertal animals, the developmental competence of in vitro-matured prepubertal oocytes is lower than that of oocytes derived from adults (for review see [18]). Prepubertal ovine oocytes show some structural and functional limitations compared to the adult ones, which are at the basis of their reduced developmental competence [19–27].

The aim of our work is to verify if oocyte developmental potential is related to the timing of meiotic progression and to mitochondrial distribution and activity. We took advance of the experimental model characterized by the comparison of adult and prepubertal oocytes, which represent a model of high and low developmental competence, respectively.

Thus, we compared the kinetic of meiosis resumption after incorporation in an in vitro maturation system and the pattern of mitochondrial distribution and activity in adult and prepubertal ovine oocytes.

## Materials and Methods

The Ethics Committee of the Sassari University approved this study.

All chemicals in this study were purchased from Sigma Chemical CO (St. Louis, MO, USA) unless stated otherwise.

### Experimental design

Oocytes from prepubertal (30–40 days old) and adult (4–6 years old) ewes were obtained from ovaries collected at a commercial slaughterhouse. Thereafter, the following experiments were performed:

1. Determination of prepubertal and adult oocytes meiotic and developmental competence: prepubertal and adult oocytes were incorporated into an in vitro embryo production system in order to compare their meiotic and developmental competence, as evaluated by maturation and fertilization rates, embryo developmental kinetics and total blastocyst output.
2. Evaluation of the kinetic of meiotic progression: timing of meiosis progression is considered as a marker of oocyte quality [16]. Therefore, we compared the progression of meiotic maturation between adult and prepubertal oocytes at different time points after oocyte incorporation in the in vitro maturation system: i) GV-MI transition was evaluated after 6,7, 8 and 9 hrs of in vitro culture; ii) MI-MII transition was evaluated after 19, 20, 21 and 22 hrs of in vitro culture. At each time point, in three replicates, at least 20 prepubertal and adult oocytes were retrieved from the maturation system and processed for nuclear staining.
3. Determination of protein electrophoretic pattern during meiosis: it has been proposed that protein synthesis and secretion are related to developmental competence of mammalian oocytes [28]. We compared protein electrophoretic pattern of prepubertal and adult oocytes at different time points during in vitro maturation: 0, 7, 19 and 24 hrs after culture. At each time point, 20 prepubertal and adult oocytes were retrieved from the maturation system and processed for 2D-electrophoresis.
4. Quantification of ATP intracellular content: oocyte energetic level has been regarded as a suitable marker of its quality since during meiotic progression an increase in ATP content is required for subsequent fertilization and embryo development [6, 8, 29]. We determined ATP intracellular content in prepubertal and adult oocytes at different time points after incorporation in an in vitro maturation system: 0, 7, 19 and 24 hrs after culture. At each time point, in three replicates, 20 prepubertal and adult oocytes were retrieved from the maturation system and processed for ATP intracellular content determination.
5. Submicroscopical evaluation of mitochondria and organelles by light and transmission electron microscopy: cumulus oocyte complexes (COCs) collected in three replicates from prepubertal and adult ovaries were processed at 0, 7, 19 and 24 hrs of IVM.
6. Evaluation of active mitochondrial distribution patterns: COCs collected in three replicated experiments from prepubertal and adult ovine ovaries were processed at collection (0 hrs) and after 24 hrs of IVM to evaluate mitochondrial distribution, mitochondrial activity and chromosomal configuration and then analyzed by confocal laser scanning microscopy (CLSM).

### Experiment 1: Determination of prepubertal and adult oocyte meiotic and developmental competence

Collected ovaries were transported from the commercial slaughterhouse to the laboratory within 1–2 h in Dulbecco Phosphate Buffered Saline (PBS) with antibiotics at 27°C. After being washed in PBS fresh medium, the ovaries were sliced using a micro-blade and the follicle content was released in medium TCM199 (with Earle’s salts and bicarbonate) supplemented with 25 mmol HEPES, 0.1 g/L penicillin, 0.1 g/L streptomycin and 0.1% (w/v) polyvinylalcohol (PVA). Cumulus—oocyte complexes (COCs) with 4–10 layers of granulosa cells, oocytes with a uniform cytoplasm, homogenous distribution of lipid droplets in the cytoplasm and outer diameter of about 90 μm (mean) were selected for these experiments. COCs selected for in vitro culture were washed three times in the same fresh medium and matured in vitro in TCM 199 supplemented with 10% heat-treated oestrus sheep serum (OSS), 1 IU/mL of FSH/LH, 8 mg/mL pyruvate and 100 μM cysteamine. 40–45 COCs were put in 500 μL of maturation medium in four-well Petri dishes (Nunclon, Nalge Nunc International, Denmark), layered with 300 μL of mineral oil and cultured for 24 h in 5% CO_2_ in air at 39°C.

Meiotic competence was determined in 4 replicated experiments in prepubertal (n = 1357) and adult (n = 970) oocytes. After 24 hrs of in vitro maturation COCS were denuded of cumulus cells using a narrow bore glass capillary, fixed for 24 h in acetic acid ethanol solution (1:3 vol/vol), and stained with 1% (wt/vol) of Lacmoid. Chromatin configuration was evaluated under a phase contrast microscope at 200 to 400 x.

Fertilization potential was computed in 3 replicates using 1070 prepubertal and 766 adult in vitro matured oocytes. COCs were partially stripped of the granulosa cells and fertilized in vitro at 39°C and 5% CO_2_, 5% O_2_ and 90% N_2_ atmosphere in four-well Petri dishes (Nunclon).

Frozen thawed spermatozoa from the same ejaculate of a single ram were used for all experimental procedures. The IVF system was composed of 300 μL of synthetic oviductal fluid (SOF) medium supplemented with 2% heat-treated ovine oestrus serum (OSS) and swim-up derived motile spermatozoa, at 1x10^6^ spermatozoa/mL concentration, layered with mineral oil. After 14 hrs oocytes were washed from spermatozoa and fixed with acetolacmoid as described above. Oocytes were classified as fertilized when showed 2 pronuclei, as parthenogenetic when showed only one pronucleus and as polyspermic when presented more than 2 pronuclei or one pronucleus and 2 or more decondensed sperm nuclei.

Developmental competence was determined in 5 replicated experiments after in vitro maturation of prepubertal (n = 2110) and adult (n = 1522) oocytes. Fertilization was carried out as above described and at 24, 26, 32 hrs post-insemination the number of cleaved oocytes, showing two distinct blastomeres, was recorded. Total cleavage computed all oocytes which cleaved within 32 hrs post insemination. The cleaved embryos were transferred to a culture system composed by SOF medium supplemented with 4 mg/mL of bovine serum albumin and essential and non-essential amino acids at oviductal concentration, and kept in maximum humidified atmosphere, at 39°C, 5% O_2_, 5% CO_2_. Newly formed blastocysts were recorded daily, starting from the 6^th^ day of culture. Total blastocysts computed those obtained within 9 days post insemination.

### Experiment 2: Evaluation of the kinetic of meiotic progression

Oocyte in vitro maturation was performed as above described. In three replicates, at each time point (0, 6 to 9, 19 to 22 hrs of in vitro culture), oocytes were decumulated by gentle pipetting using a narrow bore glass capillary, fixed in ice cold methanol, and incubated with 10μg/mL Hoechst 33342 in ice-cold methanol for 15 min. Stained oocytes were mounted into a small droplet of glycerol on a glass slide and examined under an epifluorescence inverted microscope (Nikon Diaphot, Japan).

Here and thereafter, 0 hrs of maturation indicate GV stage, 7 hrs indicate MI stage and 22 or 24 hrs indicate MII stage.

### Experiment 3: Determination of protein electrophoretic pattern during meiosis

Prepubertal and adult COCs were matured as described above, retrieved from the culture system at different time points (0 hrs, 7 hrs, 19 hrs and 24 hrs), denuded by gentle pipetting and stored at -80°C until analysis. At thawing, oocytes (n = 20 from each experimental group) were diluted to 300 μl with rehydration buffer (8M urea, 4% CHAPS, 20 mg/ml DTT, 2% pharmalytes, and trace amount of bromophenol blue) and used to rehydrate a pH 3–10 linear gradient IEF Immobiline Dry Strip (Biorad, Hercules, CA, USA). Isoelectric focusing (IEF) was performed using a Protean IEF Cell (Biorad) for 12 hrs at 20°C, followed by 500V for 1,000 Vhr, 1,000V for 2,000 Vhr, and 8,000V for 22,000 Vhr.

IPG strips were incubated in equilibration buffer 1 (50mM Tris—HCl, pH 8.8, containing 6M urea, 30% glycerol, 2% SDS, trace amount of bromophenol blue, and 100 mg/ml DTT) for 15 min, rinsed with equilibration buffer 2 (50mM Tris—HCl, pH 8.8, containing 6M urea, 30% glycerol, 2% SDS, trace amount of bromophenol blue, and 450 mg/ml iodoacetamide) for 10 min. Strips were rinsed in SDS—gel running buffer, transferred into 12% SDS gels and sealed with 0.5% (w/v) agarose solution before running the second dimension (50 mA/gel) in a 15°C termostated Protean II electrophoretic cell (Biorad). After electrophoresis, gels were silver stained (SilverQuest staining kit, Invitrogen, USA) and dried (Biorad Gel dryer). Experiments were repeated three times and differences between gel images were analyzed using the PdQuest software (Biorad). This software allows us to compare gels obtained from the two experimental groups at the same maturation time, in order to identify the spots present in gels of only one experimental group (qualitative differences), and to determine differences in intensity (quantitative differences) between spots present in gels of both groups at the same maturation time.

### Experiment 4: Quantification of ATP intracellular content

ATP was quantified in oocytes using capillary electrophoresis according to Zinellu et al. [30]. After being picked up from maturation systems at different hours and decumulated by gentle pipetting, oocytes were assessed for chromatin configuration after Hoechst staining, under an epifluorescence microscope (Nikon, Diaphot) at 40x magnification [31]. At each time point 20 oocytes showing a specific nuclear stage were selected: i.e. GV decondensed chromatin in germinal vesicle at 0 hrs, MI (metaphase plate) at 7 hrs and MII (metaphase plate and a polar body at 19h and 22h of maturation. Selected oocytes were transferred in 5 μL PBS, mixed with 5 μL of ice cold 0.6 mol/L perchloric acid and incubated for 15 min at room temperature before being centrifuged for 3 minutes at 10000 rpm in an Eppendorf Microfuge (Eppendorf, Hamburg, Germany). The supernatant was neutralized with 1.5 μL of 3.5 mol/L K_2_CO_3_. After 3 minutes centrifugation in a Eppendorf Microfuge at 10000 rpm, the supernatant was derivatized before capillary electrophoresis. Ten μL of sample or standard were mixed with 40 μL of 1.8 mol/L 1-ethyl-3-(3′-N,N′-dimethyl-aminopropyl)-carbodiimide hydrochloride (dissolved in 50 mmol/L HEPES buffer, pH 6.5) and 5 μL of 27 mmol/L Bodipy FL EDA (dissolved in 50 mmol/L HEPES buffer, pH 6.5) and incubated for 25 h at 37°C in the dark. A P/ACE 5510 CE system equipped with Laser Induced Fluorescence (Beckman instruments, CA, USA) was used. The dimension of the uncoated fused silica capillary was 75 mm ID and 57 cm length (50 cm to the detection window). Analysis was performed applying 21 nl of sample under nitrogen pressure (0.5 psi) for 3 s using a 10 mmol/L sodium phosphate buffer, pH 11.4. The separating conditions (22 kV at normal polarity) were reached in 20 s and held at a constant voltage for 8 min. All separations were carried out at 40°C.

### Experiment 5: Submicroscopical and morphometric evaluation of mitochondria and organelles by light and transmission electron microscopy

Prepubertal (n = 82) and adult (n = 68) COCs were fixed at sampling (0 hrs), and at different time intervals during IVM (7, 19, 24 hrs), and processed for light microscopy (LM) and transmission electron microscopy (TEM) analysis as previously described [32]. COCs fixation was performed in 1.5% glutaraldehyde (SIC, Rome, Italy) in PBS solution. After fixation for 2–5 days at 4°C, the samples were rinsed in PBS, post-fixed with 1% osmium tetroxide (Agar Scientific, Stansted, UK) and rinsed again in PBS. Oocytes were then embedded in small blocks of 1% agar of about 5×5×1 mm in size, dehydrated in ascending series of ethanol (Carlo Erba Reagenti, Milan, Italy), immersed in propylene oxide (BDH Italia, Milan, Italy) for solvent substitution, embedded in epoxy resin EMbed-812 (Electron Microscopy Sciences, Hatfield, PA, USA) and sectioned by a Reichert-Jung Ultracut E ultramicrotome. Semithin sections (1 μm thick) were stained with toluidine blue, examined by LM (Zeiss Axioskop) and photographed using a digital camera (Leica DFC230). Ultrathin sections (60–80 nm) were cut with a diamond knife, mounted on copper grids and contrasted with saturated uranyl acetate followed by lead citrate (SIC, Rome, Italy). They were examined and photographed using a Zeiss EM 10 and a Philips TEM CM100 Electron Microscopes operating at 80KV. Semithin sections and ultrathin sections were used also for a morphometric evaluation in all groups of the number of mitochondria, number of mitochondrial clusters and number of mitochondria per each cluster at sampling (0 hrs) and after 24 hrs of IVM.

The number of mitochondrial clusters was evaluated at the LM level in 5 oocytes per group, on at least 3 equatorial sections per oocyte (distance between the sections: 3–4 μm), and values were expressed in number of clusters per 100 μm^2^ of the oocyte area. Mitochondrial counting was performed through collection of low-magnification TEM microphotographs of the same oocytes, on 3 equatorial sections per oocyte. Images were further enlarged on the PC screen, in order to easily recognize and count mitochondria. Values were expressed in number of mitochondria per 100 μm^2^ of the oocyte area. The number of mitochondria per each cluster was extrapolated from an integrated evaluation of the above data.

### Experiment 6: Evaluation of mitochondrial activity and active mitochondrial distribution by confocal laser scanning microscopy

COCs collected from prepubertal (n = 45) and adult (n = 41) ewes ovaries in three replicate experiments were processed at collection (0 hrs) and after 24 hrs of IVM to evaluate the mitochondrial distribution, mitochondrial activity and chromosomal configuration and then analyzed by confocal laser scanning microscopy (CLSM). COCs were subjected to a double staining with MitoTracker Red CM-H_2_XRos (Molecular Probes, Inc., Eugene, OR, USA; M-7513), hereafter called MT-Red, a mitochondrial-specific fluorescent and cell-permeant probe and Hoechst 33342, to stain chromosomes. Reduced MT-Red is a derivative of dihydro-X-rodamine and is readily sequestered only by actively respiring organelles, depending upon their oxidative activity. This reduced probe does not fluoresce until it enters live cells, where it is oxidized to the corresponding fluorescent mitochondrion-selective probe and then sequestered in the mitochondria.

Immature germinal vesicle (GV) stage oocytes were retrieved at 0h IVM and used as controls. Following IVM, COCs were incubated for further 30 min at 38.5°C in TCM-199 plus 10% FCS supplemented with 1 IU/mL FSH,1IU/mL LH, 100 μM cysteamine, 8 mg/mL pyruvate; 500 nM MT-Red (stock solution: 100 μM in DMSO).

After exposure of COCs to the probes, cumulus cells were mechanically removed from the oocytes by repeated pipetting. Oocytes were then washed three times in fresh, pre-warmed 0.1% PVA in PBS/ and fixed in 2.5% glutaraldehyde/PBS for at least 15 min. After fixation, oocytes were washed three times in PBS/PVA and mounted on glass slides with 2.5 μg/mL Hoechst 33342 3:1 (v/v) in PBS and glycerol solution. Slides were kept at 4°C in darkness until evaluation.

A Leica TCS SP5 CLSM with LAS lite 170 Image software equipped with a 405-nm diode laser and a multiphoton laser was used for chromosomal and mitochondrial analysis. For mitochondrial evaluation, samples were observed with a multiphoton laser to detect MitoTracker Red CM-H_2_XRos (ex: 579 nm; em: 599 nm). Each oocyte was examined along the z-axis by means of about 30 serial confocal planes (each 3 μm thick), going from the top to the bottom of the cell. Microscope adjustments and photomultiplier settings were kept constant for all experiments. Acquisition, storage and image analysis were made with the LAS lite 170 Image software. To evaluate quantitatively the fluorescence intensity in the confocal microscopic sections an image analysis was performed using ImageJ software (V, 1.43u; National Institutes of Healt; [33]).

### Statistical analyses

Maturation, fertilization and developmental rates, kinetics of maturation and development and active mitochondria phenotypes were analyzed using chi square test or Fisher exact test when appropriate. ATP concentrations, 2D electrophoretic protein spots during meiotic progression, morphometric data on semithin and ultrathin sections and fluorescence intensity data obtained by confocal microscopy were analysed by ANOVA test after analysis for homogeneity of variance by Levene’s test.

Statistical analyses were performed using the statistical software program Statgraphic Centurion XV (version15.2.06 for Windows; StatPoint, Inc., Herndon, VA, USA) and a probability of P ≤ 0.05 was considered to be the minimum level of significance.

## Results

### Experiment 1: Determination of prepubertal and adult oocyte meiotic and developmental competence

As showed in Table 1, maturation rates after 24 hrs of culture and fertilization rates did not differ between prepubertal and adult oocytes. On the other hand, prepubertal oocytes showed higher rates of both spontaneous parthenogenetic activation (17.38% vs 2.08% in adults), and polispermy (14.30 vs 2.21% in adults; P<0.01). Thereafter, the higher developmental competence of adult oocytes compared to prepubertal ones was confirmed by higher cleavage rates (67.1 vs 60.0% in adult and prepubertal oocytes respectively; P<0.05) and higher blastocyst output (51.3 vs 19.9% in adult and prepubertal oocytes, respectively; P<0.01).

**Table 1:** Meiotic and developmental competence of prepubertal and adult sheep oocytes.

			PREPUBERTAL	ADULT
Meiotic competence	metaphase II		1290 (95.1%)	938 (96.7%)
Developmental competence	fertilization	2 pronuclei	713 (66.73%)	541 (70.62%)
		1 pronucleous	186 (17.38%)^a^	16 (2.08%)^b^
		polyspermic	153 (14.3%)^a^	17 (2.21%)^b^
	cleavage	22 hpi*	206 (16.27%)^a^	361 (35.36%)^b^
		26 hpi*	311 (24.57%)^a^	345 (33.79%)^b^
		32 hpi*	749 (59.16%)^a^	315 (30.85%)^b^
		total°	1266 (60.00%)^a^	1021 (67.08%)^c^
	blastocysts	6 dpi^&^	0^a^	234 (35.36%)^b^
		7 dpi^&^	122 (24.57%)^a^	131 (33.79%)^b^
		8 dpi^&^	87 (59.16%)	159 (30.85%)
		9 dpi^&^	43 (17.06)^a^	0^b^
		Total^	252 (11.94%)^a^	524 (34.43%)^b^

Into rows different superscripts are statistically different (Chi square test: a vs b = P<0.01; a vs c = P<0.05).

* computed on the number total cleaved embryos.

^°^ computed on the number of inseminated oocytes.

^&^ computed on the number of the total blastocysts.

^^^ computed on the number of inseminated oocytes.

In addition, the kinetic of embryo development was delayed in prepubertal oocytes compared to adult ones. At 26 hrs post-fertilization, adult fertilized oocytes underwent the first cleavage at higher rate compared to prepubertal ones (69.2% vs 40.8%, respectively; P<0.01). Therefore, the first cell cycle was longer in prepubertal than in adult fertilized oocytes. This delay grew as embryo development progressed and reached 24 hrs at the blastocyst stage.

### Experiment 2: Evaluation of the kinetic of meiotic progression

**Fig 1:**
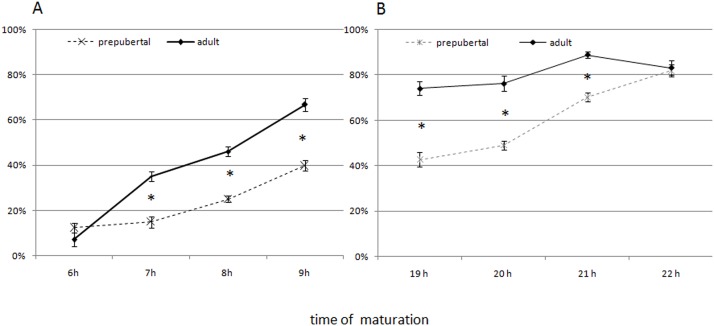
Kinetics of in vitro maturation in prepubertal and adult oocytes. Values are expressed as (A) percentages of prepubertal (n = 1186) and adult (n = 1139) oocytes that reached MI at 6 to 9 hrs of maturation culture and (B) percentages of prepubertal (n = 1026) and adult (n = 981) oocytes that reached MII stages between 19 and 22 hrs of maturation culture. *indicates statistical difference at each time point between the two experimental groups; (Chi square test: P<0.001).

The kinetic of maturation differs between adult and prepubertal oocytes. As showed in Fig 1A, oocytes derived from adult (n = 1139) and prepubertal (n = 1186) ewes reached the MI stage with a different timing. At 7 hrs of culture, MI rates were higher in adult than in prepubertal oocytes (34.7% vs 14.1% in adult and prepubertal oocytes respectively; P<0.001). Prepubertal oocytes reached similar MI rates only between 8 and 9 hrs of culture, indicating a delay in the kinetic of maturation of at least 1 hour. The delay in meiotic progression in prepubertal oocytes was confirmed by data obtained during MI-MII transition, monitored from 19 to 22 hrs of IVM. As showed in Fig 1B, the MII stage was reached earlier in adult (n = 1026) than in prepubertal (n = 981) oocytes (P<0.001). In fact at 19 hrs of culture the MII nuclear plate was observed in the 70.4% of adult oocytes compared to the 36.4% of prepubertal ones. Prepubertal oocytes reached MII rates comparable with adult ones only at 22 hrs of in vitro culture.

### Experiment 3: Determination of protein electrophoretic pattern during meiotic progression

**Fig 2:**
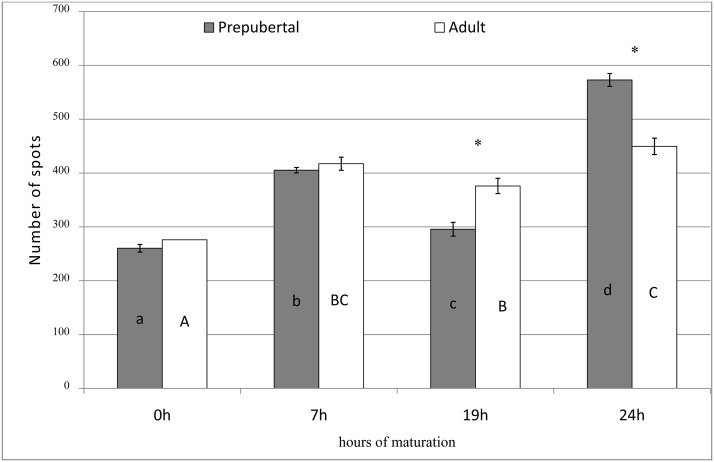
Quantification of spots detectable in 2D electrophoresis gels of proteins extracted from 20 prepubertal and adult oocytes at different times of maturation culture. (ANOVA; * indicates statistical difference between adult and prepubertal groups at each time; different upper case letters indicate statistical difference into adult group; different lower case letters indicate statistical difference into prepubertal group).

**Fig 3:**
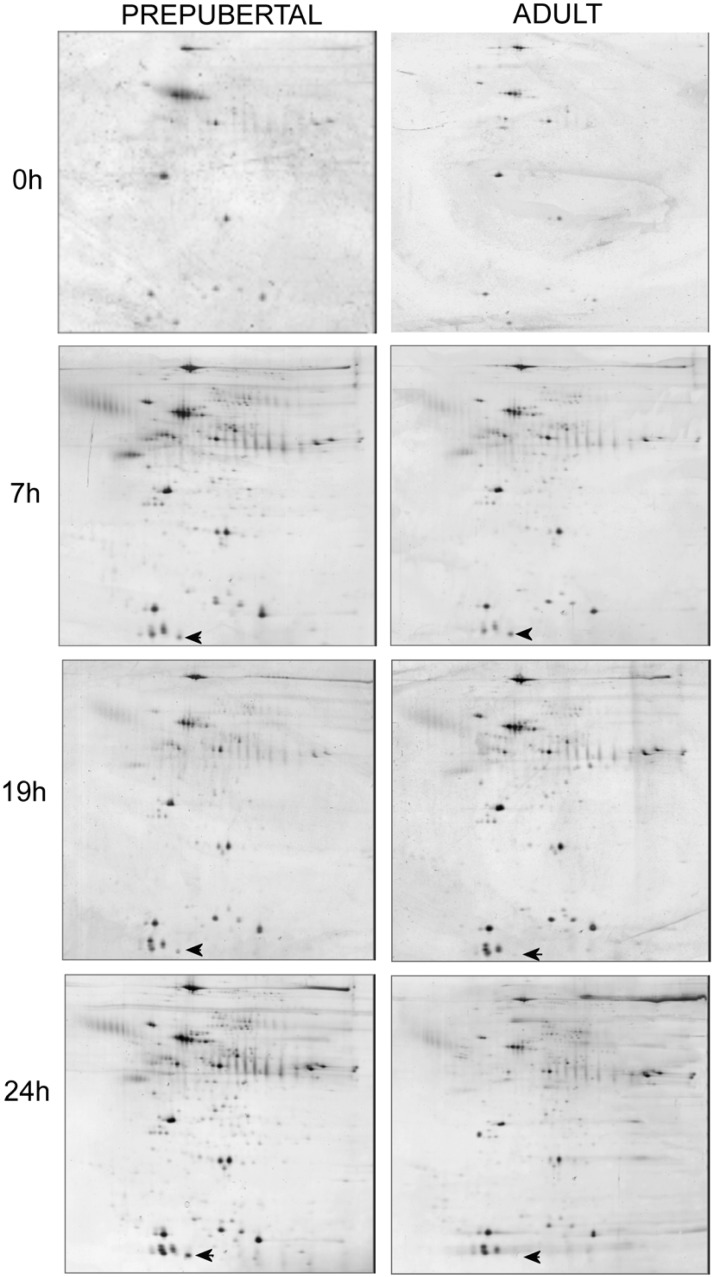
Representative electrophoretic gels of 20 oocyte proteins at 0, 7, 19 and 24 hrs of maturation culture. Arrows represent one example of a spot detectable in both prepubertal and adult oocyte protein gels at 7 hrs of maturation and detectable only in prepubertals at 19 and 24 hrs of culture.

Fig 2 resumes data on quantification of spots resolved in 2D electrophoretic gels of proteins extracted from prepubertal and adult oocytes at different time points of in vitro maturation. After 9 hrs of in vitro culture, the number of spots were statistically lower in prepubertal than in adult gels (295.6 ±12.8 vs 376±14.2 respectively; P<0.05), while at 24 hrs more spots were counted in prepubertal gels than in adults (573±11.9 vs 449.7±15.2; P<0.01). Qualitative differences in specific spots, (i.e. spots present in gels of only one experimental group at each time) after image analysis of prepubertal and adult gels are showed in Table 2. A representative panel of the electrophoretic gels of prepubertal and adult proteins at different hours of maturation is showed in Fig 3.

**Table 2:** Qualitative spot differences between prepubertal and adult 2D electrophoretic gels at different times of in vitro oocyte maturation.

Group	0h	7h	19h	24h
prepubertal	0	62	8	121
adult	25	12	82	12

In each row are resumed the number of spots present only in the electrophoretic gels of a group (prepubertal or adult) but not in the other (adult or prepubertal) at each essay time during maturation.

### Experiment 4: Quantification of ATP intracellular concentration

**Fig 4:**
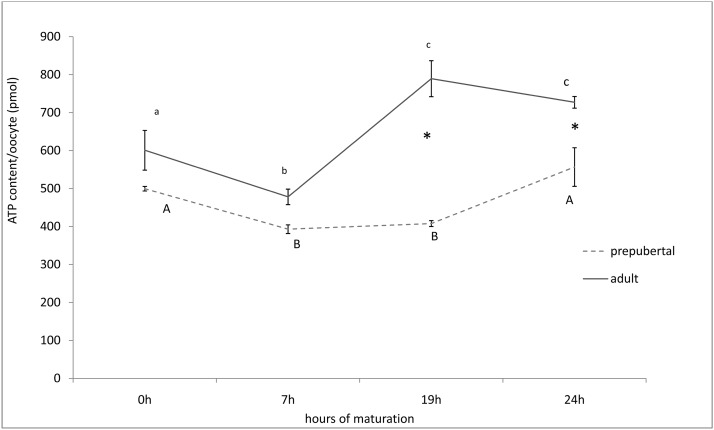
Fluctuation of ATP intracellular content in prepubertal and adult ewe oocytes during in vitro maturation. Data are expressed as mean ± SEM. Lower case letters indicate statistical difference among different time points in the adult group: ANOVA p<0.01. Upper case letters indicate statistical difference among different time points in the prepubertal group: ANOVA p<0.01. * Indicates statistical difference at each time point between adult and prepubertal groups (ANOVA: p<0.01).

As summarized in Fig 4, ATP concentration during maturation was lower in prepubertal than adult oocytes (mean ± SEM, 2.32 ± 0.09 vs 3.24 ± 0.18 pmol/oocyte respectively). In adult oocytes, after a significant decline observed from 0 to 7 hrs of culture (from 3.00 ± 0.26 pmol to 2.39 ± 0.10 pmol; P<0.01), ATP intracellular concentration peaked at 19 hrs (3.95 ± 0.24 pmol) of culture and then remained stable.

In prepubertal oocytes, a similar pattern was observed in the first 7 hrs of culture, but ATP intracellular concentration started to rise again only after 19 hrs of culture and did not reach values higher than the initial ones after 24 hrs of culture.

### Experiment 5: Submicroscopical and morphometric evaluation of mitochondria and organelles by light and transmission electron microscopy

According to Khalili and coworkers [34], type and quality of the organelles, with particular regard to mitochondria were evaluated by TEM and taken into consideration to evaluate ultrastructural differences between prepubertal and adult COCs.

#### IVM: 0 hrs (control groups)

**Fig 5:**
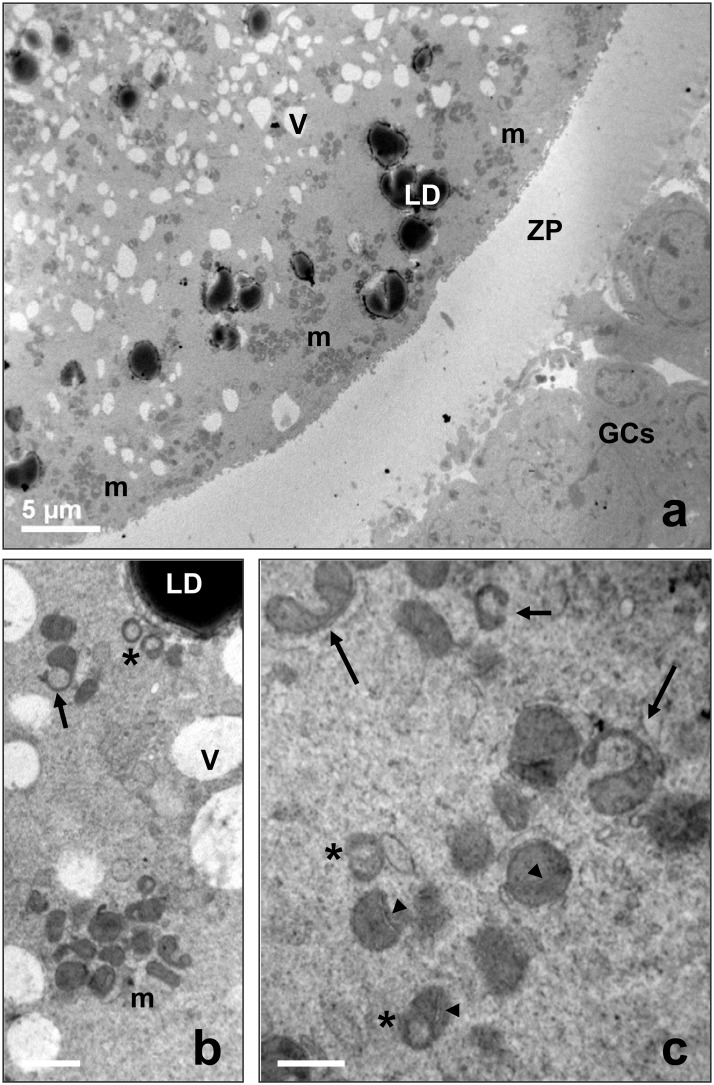
Morphology of prepubertal ovine oocytes (control group). (a) Representative micrograph by TEM, showing the oocyte surrounded by a continuous zona pellucida (ZP) and a multilayer of granulosa cells (GCs). A normal distribution of vacuoles (V) and lipid droplets (LD), typical for the ovine oocyte, is seen. Note also the presence of numerous mitochondria (m). Bar: 5 μm. (b) Rosette-like arrangement of mitochondria. TEM, bar: 1 μm. (c) High magnification TEM micrograph of mitochondria. Arrows indicated hooded mitochondria. Asterisks: mitochondria containing a clear vesicle. Arrowheads: mitochondrial cristae. V: vacuoles; m: mitochondria; LD: lipid droplets.

**Fig 6:**
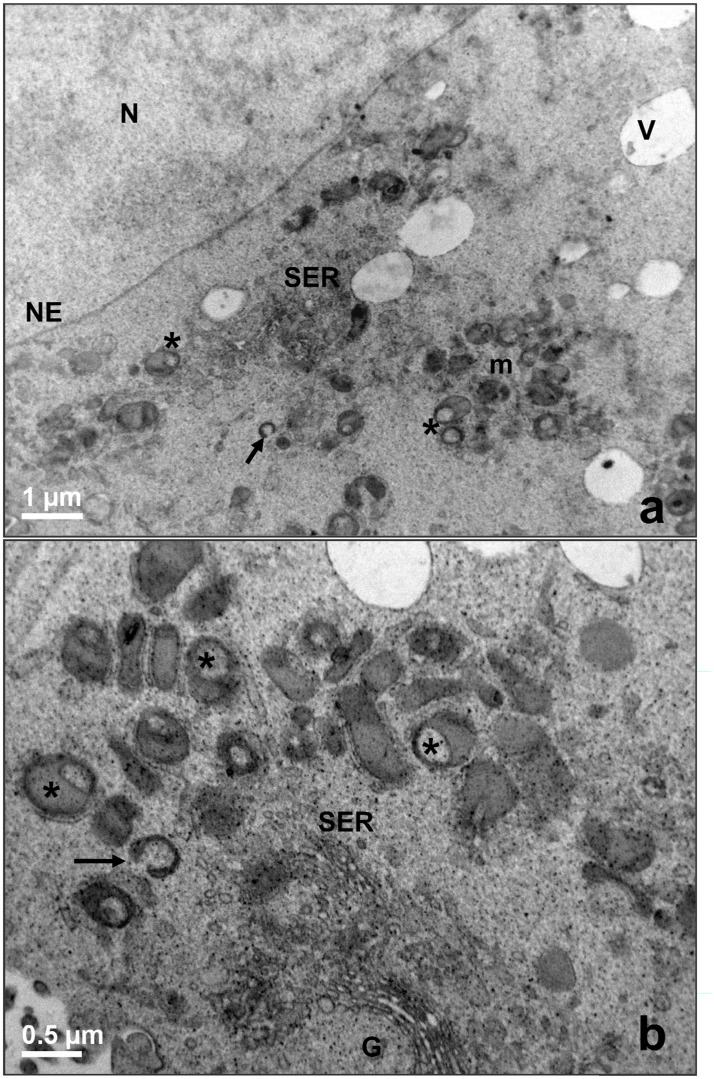
Morphology of adult ovine oocytes (control group). (a) Clusters of mitochondria (m) in close proximity to tubular element of the smooth endoplasmic reticulum (SER) and clear vacuoles (V). TEM, bar: 1 μm. (b) Groups of round-to-ovoid mitochondria (m) near to tubular elements of SER and a Golgi apparatus (G). TEM, bar: 1 μm. (c). N: nucleus, NE: nuclear envelope; V: vacuoles; m: mitochondria. Arrows: hooded mitochondria; asterisks: mitochondria containing a clear vesicle.

By LM and TEM at low magnification, all the oocytes evidenced a round shape, with a thin perivitelline space surrounded by a continuous zona pellucida. The ooplasm appeared rich of clear vacuoles and strongly electron-dense lipid droplets (Fig 5a). A rosette-type arrangement of round-to-ovoid mitochondria, interspersed among single mitochondria, vacuoles, lipid droplets and SER, was evidenced in prepubertal and adult oocytes by TEM (Figs 5 and 6). Mitochondrial clusters were often bigger in the cortical area than in the perinuclear cytoplasm. At high magnification, numerous mitochondria presented a clear vesicle inside or showed a typical hooded configuration (Figs 5b, 5c and 6). Mitochondrial *cristae* were easily discernible (Fig 5c). Well developed smooth endoplasmic reticulum (SER) and Golgi membranes were observed in adult oocytes (Fig 6).

#### IVM: 7 hrs

**Fig 7:**
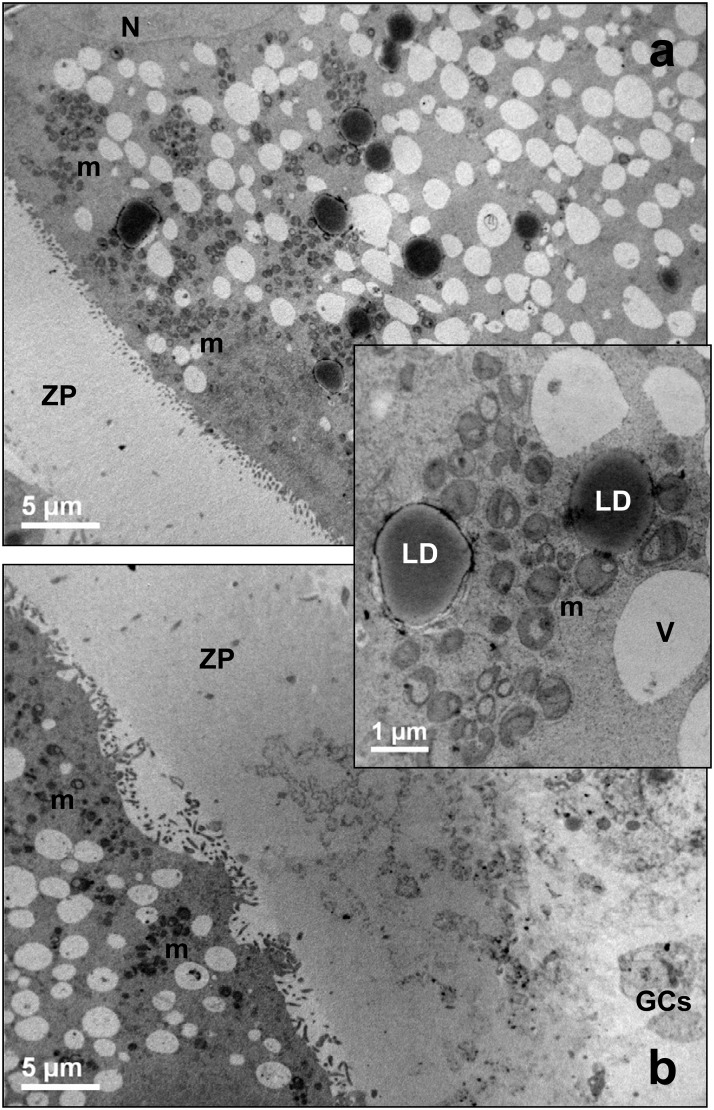
Morphology of prepubertal (a) and adult (b) ovine oocytes at 7 hrs of IVM. Representative low magnification TEM micrograph, showing the oocytes surrounded by a continuous zona pellucida (ZP), with clusters of mitochondria (m) located in the cortex. Bar: 5 μm. *Inset*: high magnification of a rosette-like arrangement of mitochondria (m). GCs: granulosa cells. ZP: zona pellucida. LD: lipid droplets. V: vacuoles. Bar: 1 μm.

The ooplasm of prepubertal and adult oocytes was rich of clusters of mitochondria dispersed among clear vacuoles of different shape and dimension, tubules of SER or elements of the Golgi apparatus (Fig 7). Mitochondrial clusters appeared sometime more prominent in prepubertal than in adult oocytes, and mostly located in the cortical ooplasm (Fig 7).

#### IVM: 19 hrs

**Fig 8:**
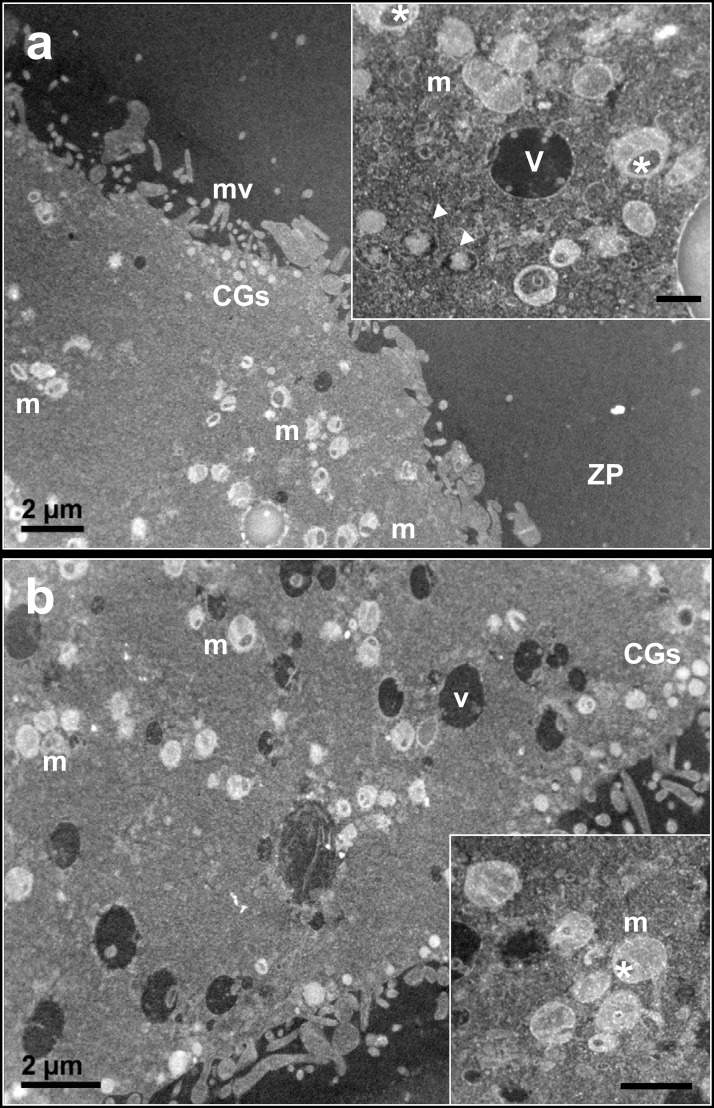
Morphology of prepubertal (a) and adult (b) ovine oocytes at 19 hrs of IVM. Representative low magnification TEM micrograph, showing the oocytes surrounded by a continuous zona pellucida (ZP), with small clusters and isolated mitochondria (m). Bar: 2 μm. *Insets*: high magnification of a small cluster of mitochondria (m). Secretory vacuoles are visible in the inset of Fig 4a (arrowheads). Bar: 0.5 μm. CGs: cortical granules; ZP: zona pellucida; V: vacuoles.

TEM analysis in prepubertal and adult oocytes showed an even distribution of small clusters of mitochondria interspersed with isolated mitochondria mainly—but not exclusively—in the cortical region (Fig 8). Aggregations of mitochondria appeared smaller than in previous sampling (IVM 0 and 7 hrs). Moreover, in prepubertal oocytes the amount of isolated mitochondria seemed to be bigger than in adults, with abundant cytoplasmic area devoid of mitochondria.

#### IVM: 24 hrs

**Fig 9:**
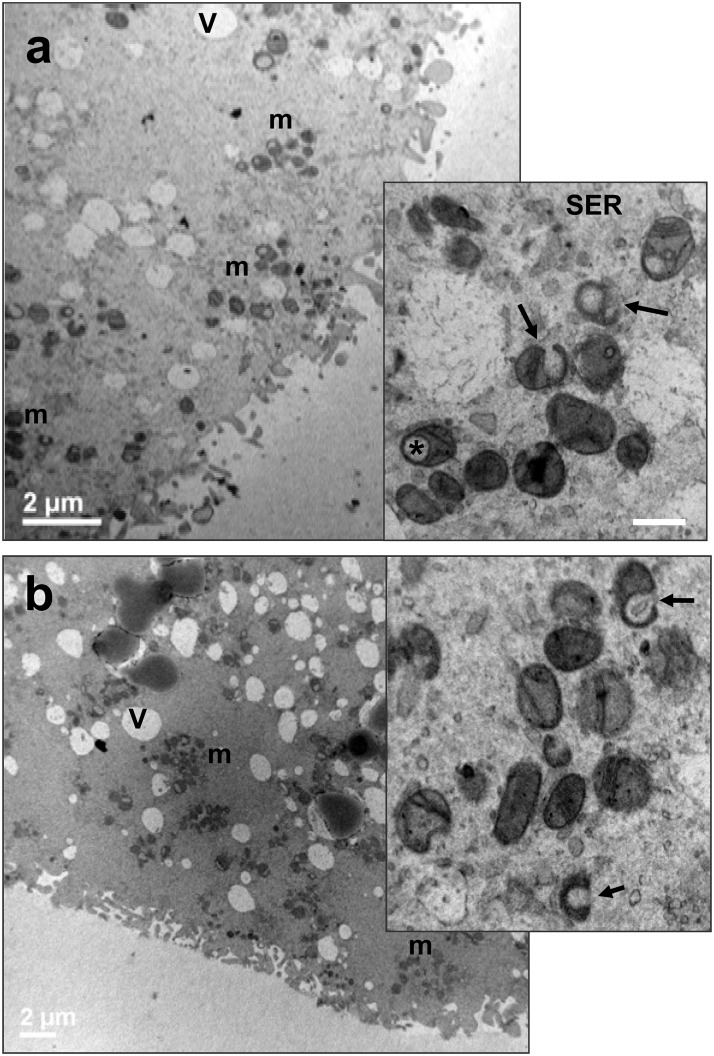
Morphology of prepubertal (a) and adult (b) ovine oocytes at 24 hrs of IVM. Representative low magnification TEM micrograph, showing the oocytes surrounded by a continuous zona pellucida (ZP), with clusters of mitochondria (m) bigger in adults than in prepubertals. Bar: 2 μm. *Insets*: high magnification of a cluster of mitochondria (m). Bar: 0.5 μm. ZP: zona pellucida; V: vacuoles. Arrows: hooded mitochondria; asterisks: mitochondria containing a clear vacuole.

At the end of the culture, a reorganization of mitochondria occurred again. Clusters appeared bigger than after 19 hrs of IVM and more voluminous in adult than in prepubertal oocytes (Fig 9). Abundant mitochondrial *cristae* were observed into hooded or ovoid mitochondria in prepubertal and adult oocytes and, often associated with membrane-bound vesicles and tubular elements of the smooth endoplasmic reticulum (*insets* of Fig 9).

#### Morphometric evaluation

Morphometric evaluation on semithin and ultrathin sections revealed that the number of mitochondria significantly increased in adult MII oocytes in comparison with the other groups, among which no significant differences were found. The number of mitochondrial clusters remains unaltered in all the groups under examination. The number of mitochondria per each cluster significantly increased only in adult MII oocytes, respect to the other groups (Table 3).

**Table 3:** Morphometric evaluation of the number of mitochondria, mitochondrial clusters and mitochondria per cluster in prepubertal and adult sheep oocytes at the GV (0 hrs of culture) and MII (24 hrs of culture) stages.

	GV prepubertal	GV	MII prepubertal	MII
		adult		adult
N° of mitochondria/100 μm^2^	26.37 ± 7.20^a^	21.25 ± 8.73^a^	29.97 ± 13.28^a^	43.19 ± 11.54^b^
N° of mitochondrial clusters/100 μm^2^	2.46 ± 0.75	2.30 ± 1.21	2.84 ± 0.93	2.94 ± 0.93
N° of mitochondria/cluster	10.04 ± 3.31^a^	8.94 ± 2.49^a^	10.21 ± 1.58^a^	14.13 ± 3.23^b^

Values are expressed as mean ± SD. Statistical analysis is calculated between columns in each row. Different superscripts in the same row indicate a significant difference (ANOVA: P <0.05).

### Experiment 6: Evaluation of patterns of active mitochondrial distribution by confocal laser scanning microscopy

**Fig 10:**
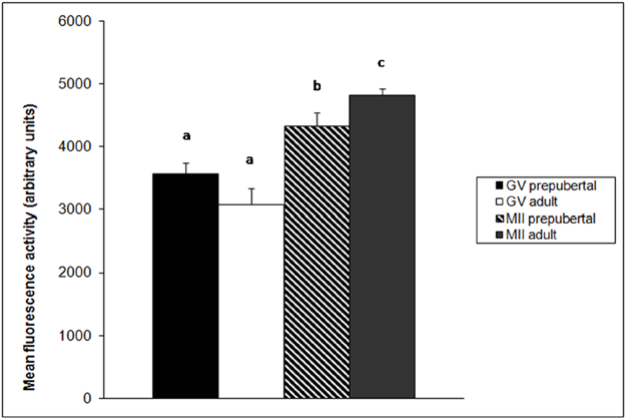
Quantification of active mitochondrial-specific fluorescence intensity of the Mitotracker stain in prepubertal and adult oocytes at GV and MII stage. Values are expressed as arbitrary units (Mean ± SE); Different letters indicate a statistical difference (ANOVA: a vs b = P<0.05; a,b vs c = P<0.01).

The fluorescent image intensity of active mitochondria measured using ImageJ software is summarized in the Fig 10. Prepubertal and adult GV stage oocytes did not shown differences in fluorescence intensity. At MII stage fluorescence is higher than in GV in both prepubertal (P<0.05) and adult (P<0.01) groups. Adult MII showed higher fluorescence compared to prepubertals.

**Fig 11:**
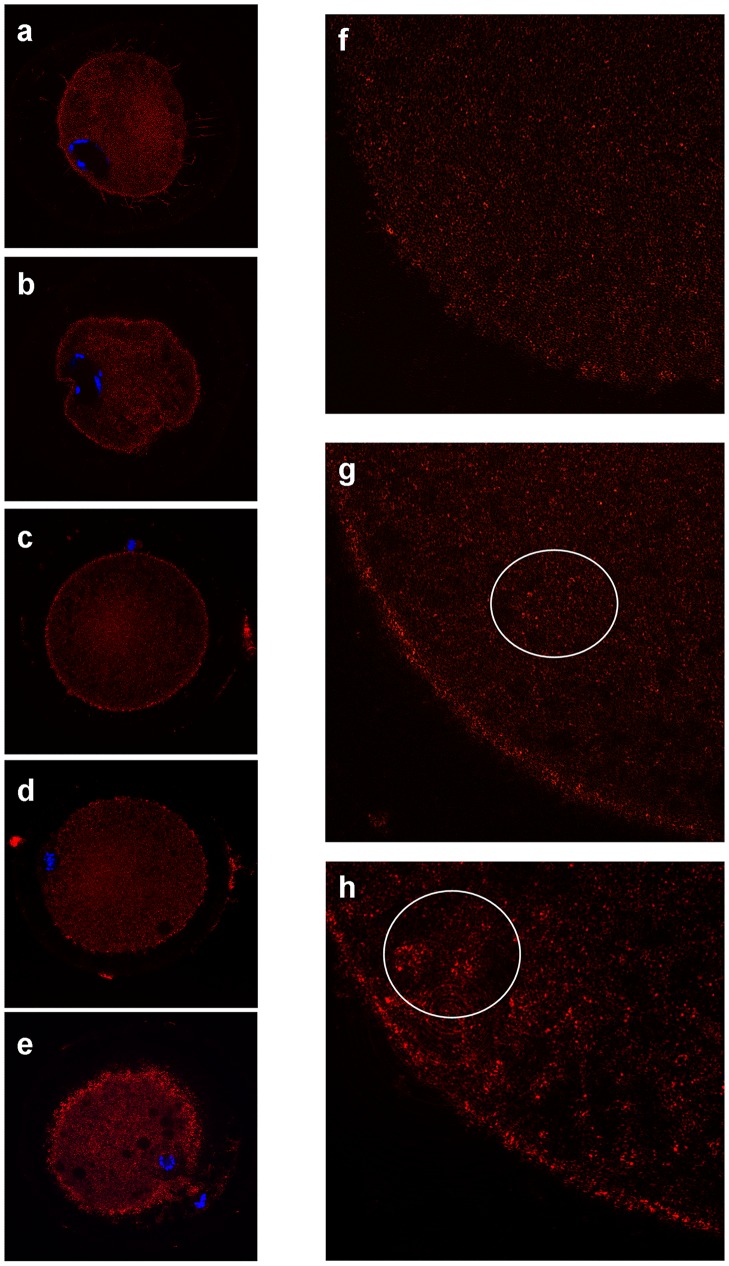
Representative CLSM patterns of active mitochondria distribution at GV and MII stages in prepubertal and adult ovine oocytes. (a) fine distribution of mitochondria in GV-stage oocytes; (b) fine distribution in MII-stage oocytes; (c) a granular distribution in GV-stage oocytes; (d) granular distribution in MII-stage oocytes; (e) clustered distribution in MII-stage oocytes. On the right are particulars of fine (f), granular (g) and clustered (h) distributions.

Mitochondrial distribution patterns were classified in three groups, as previously reported, with some modifications [35]: 1) Pattern A: homogeneous FINE, with small granulations spread throughout the cytoplasm; 2) Pattern B: homogeneous GRANULAR, with large granulations spread throughout the cytoplasm; 3) Pattern C: heterogeneous CLUSTERED, when particularly large granulations were present, spread all over the cytoplasm or located in specific cytoplasmic domains. In the majority of prepubertal oocytes observed, the distribution of mitochondria was homogenous fine (Pattern A) or granular (Pattern B), with a higher prevalence of the former (Fig 11). More specifically, Pattern A was observed in the 55.6% of prepubertal GV and 66.7% of prepubertal MII, while Pattern B in the 44.4% of prepubertal GV and 33.3% of prepubertal MII. Adult oocytes showed a different mitochondrial distribution respect to prepubertal animals, with a lower proportion of homogenous fine (Pattern A), a higher percentage of homogeneous granular (Pattern B) and the clusterization (Pattern C) only at MII stage. In detail, Pattern A was found in the 20% of adult GV and 9.1% of adult MII; Pattern B in the 80% of adult GV and 36.4% of adult MII; Pattern C in the 54.5% of adult MII stage oocytes (Table 4).

**Table 4:** Distribution of different mitochondria aggregation patterns in GV and MII prepubertal and adult ovine oocytes.

		FINE	GRANULAR	CLUSTERED
GV	Prepubertal	15 (55.6%)^a^	12 (44.4%)^a^	0^a^
	Adult	6 (20.0%)^b^	24 (80%)^b^	0^**a**^
MII	Prepubertal	18 (66.7%)^a^	9 (33.3%)^a^	0^a^
	Adult	3 (9.1%)^b^	12 (36.4%)^a^	18 (54.5%)^b^

Different superscripts are statistically different (Chi square test: P<0.01).

In prepubertal and adult GV oocytes CLSM analysis revealed that the trans-zonal cytoplasmic projections penetrating through the zona pellucida the oocyte from the surrounding granulosa cells, appeared intensely labeled by MT-Red. Moreover, an evident thin layer of active mitochondria was present under the plasma membrane in GV-stage oocytes. On the contrary, MII-stage oocytes from prepubertal and adult animals showed a more homogeneous distribution of mitochondria in the cytoplasm.

## Discussion

In the present work we showed that ATP concentration and protein pattern fluctuations in low quality oocyte is altered compared to high quality ones. We demonstrated that in GV stage of low and high quality oocyte active mitochondria have a fine or granular homogeneous distribution while only in high quality oocytes most of MII display functional mitochondria aggregated in a clustered structure. The number of mitochondria grows from GV to MII in adult oocytes but not in prepubertals, increasing the number of mitochondria/cluster. We evidence that the prompt recover of ATP production after the transition GV-MI is fundamental to oocyte progression toward meiosis and determines the kinetic of the meiotic cycle.

Present data confirm a previous work [36] showing a difference in developmental competence between prepubertal and adult ovine oocytes in terms of both quantity and quality of produced blastocysts which allow us to classify prepubertal oocytes as “low quality” oocytes. The low quality of prepubertal oocyte cytoplasm was previously associated with cytoplasmic molecular defects [24, 25, 31]. The kinetic of the first mitotic cycles has been reported to be indicative of the developmental potential of the preimplantation embryo [37, 38]. The delay in the kinetic of maturation in prepubertal compared to adult oocytes, could be related to the subsequent differences emerged in their developmental competence.

Prepubertal oocytes reach MI stage 1 hr later than adult ones. This delay grows as the first meiotic division proceeds. After 19 hrs of IVM, MII rates in adult oocytes are higher than in prepubertal ones, which reach comparable rates only after 21 hrs of culture. The delay is amplified at the blastocyst stage where the difference in development kinetic between prepubertal and adult embryos reaches almost 24 hrs. Embryonic development occurs in the absence de novo transcription till the oocyte-to-embryo transition and is dependent of mRNA and/or proteins stored into the oocyte during oogenesis [39].

Proteins appear to be of basic importance for meiotic resumption [40] but also for the attainment of cytoplasmic maturation. Abnormalities in protein metabolism during in vitro maturation were evidenced in prepubertal oocytes compared to the adult counterpart [31, 41]. We have previously shown in prepubertal oocytes alterations in the expression of some genes [24, 25], and in the ATP-dependent phosphorylation activity of key proteins involved in the control of meiotic progression [31]. Protein pattern reprogramming plays a fundamental role in the control of meiotic cycle and it is an essential process in the preparation of mammalian oocyte for fertilization. In our experiments we evidenced a perturbation of protein patterns in prepubertal and adult oocytes at different times during in vitro maturation. We showed that the number of protein spots in 2D electrophoretic gels did not differ between prepubertal and adult electrophoretic gels at 0 and 7 hrs of maturation culture. At 19 h of culture the number of protein spots is higher in adults compared to prepubertal while, on the contrary, at 24 h prepubertal gels showed a higher spot number than adult ones. This suggests abnormalities in prepubertal oocyte protein metabolism that delay the acquisition of the protein pattern which allows to progress to MII. Confirming this suggestion we demonstrated that MII stage in prepubertal oocytes is reached later than in adult ones and only after 22 hrs of culture MII rates do not differ between prepubertals and adults.

Energy in the form of ATP is critical for nuclear/cytoplasmic maturation events and for developmental potential of the embryo [7, 42, 43]. Spindle formation and chromosome behaviour depend on the expression and activity of motor proteins, which use ATP as their energy source. Once the oocyte resumes meiosis, mitochondria provide ATP for cytoskeletal and cytoplasmic organization [44]. In the present study we evidenced that ATP content in adult oocytes decreases during GV/MI transition, to rise and reach a plateau at 19 hrs. In prepubertal oocytes ATP rise is delayed and till 22 hrs do not reach levels comparable to adult ones.

TEM observations revealed that the mitochondria number and its cytoplasmic distribution change during IVM, reflecting fluctuations in ATP intracellular contents. We showed an increment in the number of mitochondria in adult oocytes at MII compared to GV while in prepubertal this number did not change.

Mitochondria distribution is modified during in vitro maturation. While at 0 hrs of IVM prepubertal and adult oocytes showed a similar arrangement of mitochondria, with big cortical clusters often in close proximity to elements of the SER or Golgi apparatus, at 7 hrs such aggregations appeared smaller in adults than in prepubertal oocytes. After 19 hrs of culture a sort of reduction/dispersion of mitochondrial clusters was evidenced, with the presence of numerous isolated elements spread throughout the ooplasm, especially in prepubertal oocytes. At the end of the culture period (IVM 24 hrs), big clusters were newly visible in adult but not in prepubertal oocytes, where small aggregations were still present. In all, numerous isolated mitochondria were scattered in the cytoplasm.

Our data are in agreement with previous studies on mammalian oocytes revealing that mitochondria are redistributed during IVM (cattle: [8, 45]; dogs: [46]; goat: [47]; horse: [48]; humans: [29, 49, 50]; mice: [51]; pigs: [3, 52]). However, the conclusions were not consistent with each other, suggesting a probably species-specific mitochondrial arrangement. For example, during IVM of horse oocytes mitochondria aggregation changed from finely distributed through crystalline to an aggregated, granulated appearance [48]. Calarco [51] reported that fully mature mouse oocytes showed obvious polarity of mitochondrial distribution. In cynomolgus monkey oocytes, mitochondria were evenly distributed throughout the cytoplasm. There was no aggregation of mitochondria around nuclear material, and their distribution did not differ between GV and MII stages [53]. This is, to our knowledge, the first study comparing mitochondrial redistribution during IVM in ovine prepubertal and adult oocytes.

Català et co-workers [42] using MitoTracker Orange CMTMRos showed in prepubertal ovine oocytes that at GV stage active mitochondria are homogeneous or peripheral distributed and at MII active mitochondria are polarized around the metaphase spindle and inside the polar body. Using confocal microscopy and the fluorescent labelling MitoTracker Red CM-H_2_XRos to label active mitochondria we did not observe a similar distribution of active mitochondria, probably caused by the different fluorescent probe used. We evidenced in GV stage a sub-cortical membrane distribution of active mitochondria, which produced the amount of ATP need to maintain molecular trafficking between corona radiata and oocyte cytoplasm [54, 55]. At MII stage, active mitochondria lost this compartmentalization and were spread all over the cytoplasm and this was believed to be an indication of cytoplasmic maturation [3, 55].

This finding was in accordance with studies carried out on other mammals such as cows, pigs and humans [8]. Our study did not reveal the accumulation of mitochondria around the nucleus as showed in other species (mouse: [51]; bovine: [8]; porcine: [3, 52]; monkey: [53]; human: [56]).

Fluorescent intensity measurement of Mitotracker labelled mitochondria revealed a higher mitochondrial activity in MII than GV stage oocytes and in adult MII compared to prepubertal ones. As stated above we classified prepubertal and adult sheep oocytes into three classes, as reported by De Los Reyes [35], according to different phenotypes in the cytoplasm distribution of active mitochondria during in vitro maturation which could be related to oocyte developmental competence. A fine homogenous dispersion of active mitochondria in cytoplasm could be an indicator of poor developmental competence, being carried from most of prepubertal GV and MII stage oocytes. Most of adult GV oocytes showed the same fine granular dispersion of active mitochondria but MII stage oocytes lost this distribution pattern and acquired a granular and, mostly, a clustered distribution. The presence of granular and clustered distribution in MII adult oocytes which showed the highest ATP levels and developmental competence, suggest that mitochondria clusterization is related to an increased mitochondria activity and a higher intracytoplasmic ATP concentration. On the contrary, the persistence of diffused unclustered mitochondria indicates their low activity and it justifies the low ATP concentration measured in prepubertal oocytes. These mitochondrial distribution patterns found by CLSM are interestingly correlated to the ultrastructural study. While CLSM analysis evidenced exclusively functionally active mitochondria, ultrastructural studies allowed i) to detect all mitochondria present in a specific sub-ooplasmic domain, independently of their metabolic state, and ii) to highlight the presence of morphological alterations, that could be related to a bio-molecular imbalance. At GV stage TEM data did not show any difference in the number and distribution of mitochondria between prepubertal and adult oocytes: mitochondria presented a clusterized rosette-type arrangement interspersed among single mitochondria. At the same stage, results on active mitochondria distribution obtained by Mitotracker staining and CLSM showed that the 55.56% of prepubertal GV and the 20% of adult GV has a homogeneous fine distribution (Pattern A) and those that remain has a granular distribution (Pattern B). Overlapping these data we can speculate that some of mitochondria in clusters seen by TEM are “switched-off”, thus resulting not active in the Pattern A distribution. In the granular distribution (Pattern B) more numerous active mitochondria are present in TEM clusters, conferring the granular aspect to CLSM images.

**Fig 12:**
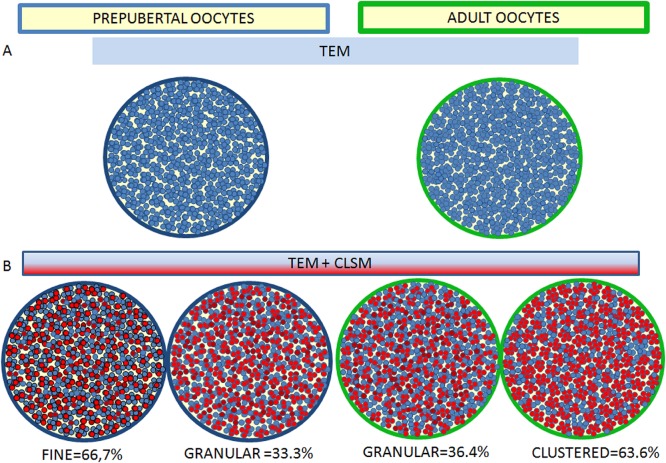
Schematic representation of data obtained by TEM and CLSM on morphological and morphometric distribution of mitochondria related to their activity in prepubertal and adult ovine oocytes. A) schematic representation of mitochondria (light blue) distribution as seen at TEM; B) superimposition of total mitochondria distribution as seen at TEM (light blue) with active mitochondria distribution as seen at CLSM (red).

At MII-stage after TEM analysis revealed a higher number of mitochondria but a comparable number of clusters in adult oocytes compared to prepubertals, meaning also a higher number of mitochondria/cluster. CLSM data evidenced that in most of prepubertal oocytes active mitochondria are finely distributed (Pattern A = 66.7%) and those that remain exhibit a granular distribution (Pattern B), while in most of adult oocytes (54.5%) active mitochondria are distributed in big clusters (Pattern C), indicating that most mitochondria of big clusters seen in TEM were active. A schematic drawing (Fig 12) depicts our morphological and morphometric evidences.

In conclusion our work evidenced the importance of the quality of oocyte cytoplasm to determine the fate of an embryo and contributed to clarify the biochemical mechanisms associated with the meiotic progression and acquisition of developmental competence. We demonstrated that mitochondria and their functional aggregation during maturation play an active role to provide energy in terms of ATP. The cytoplasmic ATP content promotes all the energy requiring processes which determine the timing of the cell cycle and the acquisition of developmental competence. The oocytes with low developmental competence have a slowed down energetic metabolism which delay physiological times of later development.
